# Temporal and geographical dynamics of early-onset Parkinson’s disease burden: insights from the Global Burden of Disease Study 2021

**DOI:** 10.3389/fneur.2025.1473548

**Published:** 2025-01-30

**Authors:** Yong Li, Dianhui Tan, Cheng Luo, Junchen Chen

**Affiliations:** The First Affiliated Hospital of Shantou University Medical College, Shantou, Guangdong, China

**Keywords:** early-onset Parkinson’s disease, Global Burden of Disease Study, epidemiology, disability-adjusted life years, socio-demographic index

## Abstract

**Introduction:**

Early-onset Parkinson’s disease (EOPD) is a rare degenerative condition of the nervous system that primarily affects individuals of working age. Its distinct clinical and genetic features make it a critical area of study in neurological research and public health.

**Methods and materials:**

This analysis utilized data from the Global Burden of Disease Study 2021, covering 371 diseases and injuries across 204 countries and territories from 1990 to 2021. The study focused on individuals aged 15–49 to characterize EOPD. Data on occurrence, frequency, mortality, and disability-adjusted life years (DALYs) were collected. Sociodemographic measures were used to analyze patterns and differences. Statistical methods, including joinpoint regression and decomposition analysis, were employed to identify temporal patterns and factors influencing variations in EOPD burden over time.

**Results:**

The global incidence of EOPD saw a significant increase between 2000 and 2009, with an average annual percentage change (AAPC) of 4.3%, continuing at a slower rate (AAPC 1.6%) from 2010 to 2021. By 2021, the incidence reached 2.1 cases per 100,000 population, up from 1.0 in 1990. Males had a higher incidence (AAPC 2.3%) compared to females (AAPC 0.8%). In 2021, 59.7% of the 81,047 global EOPD cases were male. Significant increases were observed in East Asia (AAPC 5.1%), Andean Latin America (AAPC 2.0%), and North Africa/Middle East (AAPC 1.1%), with a decline in High-income North America. China had the highest incidence in 2021 (5.17 cases per 100,000), followed by Peru and Bolivia. Saudi Arabia had the highest DALYs attributable to EOPD. The decomposition study indicated that the rise in global incidence and DALYs from 1990 to 2021 was mainly driven by epidemiological changes and population growth, with regional variations in impact.

**Conclusion:**

This global analysis highlights the need for targeted interventions and research to address gender-specific risk factors, regional disparities, and the effects of epidemiological changes on the growing EOPD burden.

## Introduction

Parkinson’s disease (PD) is a neurological condition that worsens over time and affects people’s quality of life greatly. It is defined by symptoms that affect both movement and non-movement functions (1). Early-Onset Parkinson’s Disease (EOPD) refers to a particular group of Parkinson’s disease instances that have unique clinical and genetic features and are typically identified as PD before the age of 50 (2). Due to the disease’s influence on their prime working and family-rearing years, EOPD patients frequently encounter significant obstacles, which is why neurological research and public health must prioritize EOPD (3).

Beyond just a single patient, EOPD has an international influence on families, healthcare systems, and economies all around the world. According to recent estimates, EOPD and juvenile PD make up between 5 and 15% of all PD cases, which translates to a sizable number of people considering PD’s rising incidence worldwide (4). The lengthy course of the disease, the higher probability of genetic variables, and the possibility of early intervention and management measures highlight the public health implications of EOPD.

Several epidemiological investigations have looked into the genetic mutations linked to EOPD. Studies have concentrated on mitochondrial dysfunction and gene mutations. Hedrich et al. (5), for example, reported that in patients with EOPD, DJ-1 mutations were less common than Parkin mutations. Wu et al. (6) found that EOPD patients had a greater prevalence of glucocerebrosidase gene mutations. Vela et al.’s (7) study examined the frequency of impulse control behaviors in patients with EOPD, contrasting it with that of healthy controls and examining any correlations with the use of dopaminergic drugs.

Despite these advancements, there is still a significant lack of understanding regarding the extended-term trends and global distribution of EOPD. The majority of previous research focused on point prevalence or short-term incidence, which left out important information on how the burden of EOPD is changing over time. Comprehensive international comparisons have also been hampered by the absence of uniform data collecting and reporting practices among nations (4, 8, 9).

Utilizing information from the 2021 Global Burden of Disease Study, our research addressed the significant lack of data on temporal and regional shifts in EOPD burden. We will look at worldwide trends from 1990 to 2021, pinpoint regional trends and differences, and evaluate how socioeconomic characteristics and the burden of EOPD vary by location. This study will support data-driven decision-making in worldwide health policy and allocation of resources for this important group of Parkinson’s disease patients, providing new perspectives on the evolving burden of EOPD and directing specific public health interventions and research agendas.

## Materials and methods

### Research participants and gathering of data

Analysis of the GBD 2021 was conducted using multiple cross-sectional datasets obtained from the global Health Data Exchange (GHDx). The Global Health Data Exchange contains data on the global impact of 371 illnesses and traumas in 204 countries and regions from 1990 to 2021 (10), which includes Parkinson’s disease. The GBD data collection employs a systematic approach that integrates various health data sources, such as national health surveys, vital registration systems, and disease registries, to produce reliable health metrics. This process uses statistical models, including linear and non-linear mixed effects models, to estimate correction factors based on pairs of estimates with the same demographic parameters. For example, past-year prevalence estimates of anxiety disorders are adjusted to reflect point prevalence using matched data on age, sex, location, and year (10, 11). The GBD methodology further includes adjustments for data not reported by sex or age, utilizing within-study sex ratios and age-sex splitting techniques for accurate representation. Despite these methods, the GBD data is limited by measurement errors and reporting inaccuracies due to flawed methodologies and potential underreporting of illnesses, highlighting the need for ongoing improvement in data collection systems. While GBD aims to capture uncertainty across data types and processes, fully disentangling all sources of uncertainty remains challenging. The estimation of Years Lived with Disability (YLDs) is particularly affected by data sparsity and inability to reflect variations in treatment access. The GBD 2021 cycle has enhanced cause of death data processing, including updates to Bayesian algorithms for noise reduction and adjustments for stochastic variation, ensuring that real trends are preserved even in small samples (12). Additionally, the methodology has been extensively peer-reviewed with a focus on improving transparency and accuracy in health estimates, as outlined in the Guidelines for Accurate and Transparent Health Estimates Reporting (GATHER) statement. Overall, GBD data is crucial for understanding global health trends, but it is important to recognize its limitations and ongoing efforts to refine methodologies.

Individuals younger than 50 years old are at risk for developing early-onset Parkinson’s disease (EOPD), a form of Parkinson’s disease (8). Early-onset Parkinson’s disease (EOPD) includes a rare form known as Juvenile Parkinson’s disease, which presents with symptoms and signs of Parkinson’s before the age of 21 (13). In this study, we used the age range of 15 to 49 years to offer a comprehensive explanation and widespread understanding of EOPD. The GBD research identified 21 clusters of countries that are close in location and have similar health patterns, which were utilized to gather information on Parkinson’s disease in individuals of all genders and those aged 15–49 (10).

The socio-demographic index (SDI), a composite measure of the social and economic factors that affect health outcomes globally, was computed for each nation as part of the GBD 2021 project. The calculation of SDI involves lagged income per person, average years of education for individuals aged 15 and above, and indices of total fertility between 0 and 1 for individuals under 25 (10). Zero represents the maximum fertility rate, the minimum per capita income, and the lowest level of education. The SDI consists of five quintiles: low, low-middle, middle, high, and high-middle.

We obtained data on the number of new cases, existing cases, fatalities, DALYs, frequency of occurrence, and rate of occurrence from GBD 2021. The GBD method utilized the 25th and 975th estimates out of the 1,000 estimates in order to generate 95% uncertainty intervals (UIs) (10).

Because the GBD is an openly available database, ethical approval was not required for this study with human participants, in accordance with institutional and local rules. Participants or their legal guardians/next of kin were not needed to provide written informed consent in order to comply with national legislation and institutional standards.

## Statistical analysis

This research examined the worldwide patterns in the occurrence, frequency, death rate, and disability-adjusted life years of early-onset Parkinson’s disease. The linear regression method was employed to calculate the age-specific rates and their average annual percentage changes (AAPCs). The dependent variables were rates on a logarithmic scale, while the independent variables were each year. AAPCs are calculated as weighted averages of annual percentage changes (APCs) and offer a summary of trends over multiple years (14). They enable us to compare APCs over time using a singular number. Geometrically weighted means of yearly percentage change figures were utilized for computing APCs in regression analysis. In other terms, a 0.2 AAPC would suggest a 0.2% increase in annual rates based on annual percentage changes. The trends in rates are reflected in the AAPC values and their 95% CIs. AAPCs were calculated for the time frames of 1990–1999, 2000–2009, 2010–2021, and 1990–2021.

Subsequently, we endeavored to determine the year that experienced the most significant shift in trends. To identify these trends over time, we analyzed the data using Joinpoint Regression Analysis, which is a statistical method that detects significant changes in trends, allowing researchers to identify points (joinpoints) where the trend in the data changes significantly, indicating shifts in the underlying processes affecting the data. This analysis begins by fitting the data to a basic linear regression model on a logarithmic scale, employing the Monte Carlo permutation technique to assess the significance of adding additional joinpoints, starting with a model that has no joinpoints (15, 16). The final model is selected using the Weighted Bayesian Information Criteria (WBIC) to balance model fit and complexity. Key steps in this process include initial model fitting, significance assessment through Monte Carlo permutation, and model selection using WBIC. In contrast, decomposition analysis is a statistical technique that breaks down changes in health metrics-such as incidence, prevalence, mortality, and DALYs-into components attributable to various factors, helping to understand the contributions of factors like aging, population growth, and epidemiological changes to the overall burden of a disease (17). In the context of EOPD, this analysis visualizes how changes in morbidity and mortality rates are influenced by demographic shifts and other factors, typically adjusting rates for population and age to isolate these variables’ effects on observed trends. Key steps in decomposition analysis include defining epidemiological variations, analyzing contributions of different factors, and visualizing results to highlight their relative contributions to changes in health metrics over time.

Global trends were stratified by age group, sex, and SDI, in addition to reporting on regional and national trends. The identical approach of AAPCs was utilized for analyzing and presenting the outcomes of statistical analyses, encompassing effect magnitudes and confidence intervals, proportions, uncertainty intervals, and exact *p*-values, as noted earlier (18, 19). The role of the three factors influencing incidence, prevalence, mortality, and DALY changes between 1990 and 2021 by sex difference was visualized using a decomposition analysis (i.e., aging, population, and epidemiology). Epidemiological variations are defined as morbidity and mortality rates that have been adjusted for population and age (20). R version 4.4.0, GraphPad Prism (version 9.0), and Joinpoint Regression Program (version 5.0.2) were utilized for all statistical analyses in this study.

## Results

### Global trends in EOPD population

**Figure 1:**
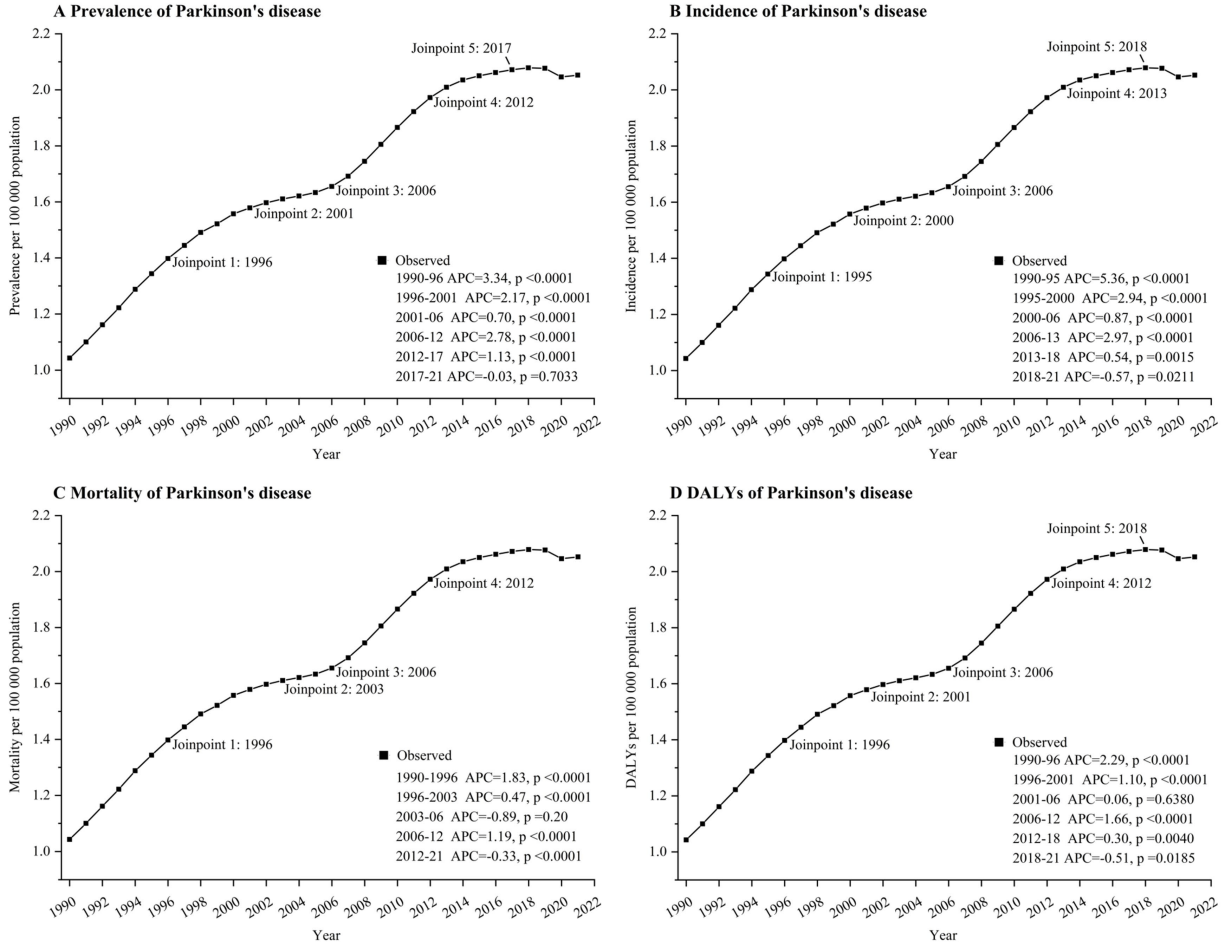
Joinpoint regression analysis of global Parkinson’s disease prevalence **(A)**, incidence **(B)**, mortality **(C)**, and DALYs **(D)** in aged 15–49 years from 1990 to 2021. APC, annual percentage change. DALYs, disability-adjusted life-years.

The prevalence of EOPD experienced a worldwide rise from 1990 to 1999 (AAPC 2.2 [95% CI 2.1 to 2.3]), followed by a swift escalation from 2000 to 2009 (AAPC 4.3 [4.1 to 4.5]), and a sustained growth from 2010 to 2021 (AAPC 1.6 [1.42 to 1.7]) (Table 1). Overall, the prevalence of EOPD in 2021 (2.1 per 100,000 individuals [95% UI 2.8 to 1.5]) exceeded that of 1990 (1.0 per 100,000 individuals [1.4 to 0.8]; AAPC 0.9 [95% CI 0.72 to 1.1]) (Table 2). Despite the fact that the prevalence of EOPD and DALYs increased between 2010 and 2021, they were not as high as they were in 1990. Nevertheless, there was a decrease in EOPD mortality from 2010 to 2021 (AAPC-0.1[−0.2 to 0.1]) (Table 1). The joinpoint analysis of EOPD incidence, prevalence, mortality, and DALYs is illustrated in Figure 1. The joinpoint regression analysis revealed a significant increase in the incidence of EOPD in 1995, 2000, 2006, 2013, and 2018 (Figure 1).

**Table 1:** Global AAPCs in prevalence, incidence, mortality, and DALYs of Parkinson’s disease.

Sex	Year	Incidence			Prevalence			Mortality			DALYs		
		AAPC (95% CI)	*t*	*p*-value	AAPC (95% CI)	*t*	*p*-value	AAPC (95% CI)	*t*	*p*-value	AAPC (95% CI)	*t*	*p*-value
Male	1990–1999	4.4(4.2 to 4.6)	52.3	<0.001	2.9 (2.8 to 3)	61.7	<0.001	1.6 (1.1 to 2.1)	6.8	<0.001	2.0 (1.9 to 2.2)	29.6	<0.001
	2000–2009	1.7 (1.6 to 1.9)	24.5	<0.001	1.6 (1.5 to 1.8)	27.5	<0.001	0.4 (0.2 to 0.5)	4.7	<0.001	0.9 (0.7 to 1.0)	10.0	<0.001
	2010–2021	0.9 (0.7 to 1.1)	10.2	<0.001	1.0 (0.9 to 1.1)	17.0	<0.001	−0.01(−0.11 to 0.09)	−0.2	0.82	0.3 (0.2 to 0.5)	4.0	<0.001
	1990–2021	2.3 (2.2 to 2.4)	46.8	<0.001	1.8 (1.8 to 1.9)	53.7	<0.001	0.7 (0.5 to 0.8)	8.2	<0.001	1.1 (1.0 to 1.2)	21.4	<0.001
Female	1990–1999	2.1 (1.9 to 2.2)	31.6	<0.001	3.0 (2.9 to 3.2)	44.4	<0.001	0.9 (0.8 to 1.1)	11.8	<0.001	1.6 (1.5 to 1.7)	32.7	<0.001
	2000–2009	4.1 (3.9 to 4.3)	43.2	<0.001	1.3 (1.2 to 1.4)	24.7	<0.001	−0.2(−0.5 to 0.2)	−0.8	0.43	0.5 (0.2 to 0.8)	3.7	<0.001
	2010–2021	1.4 (1.2 to 1.5)	17.9	<0.001	1.0 (0.9 to 1.1)	14.5	<0.001	−0.2(−0.3 to-0.2)	−6.0	<0.001	0.2 (0.1 to 0.4)	3.5	<0.001
	1990–2021	0.8 (0.6 to 1.1)	62	<0.001	1.8 (1.7 to 1.8)	45.3	<0.001	0.2 (0.1 to 0.3)	2.3	0.02	0.8 (0.7 to 0.9)	14.7	<0.001
Both	1990–1999	2.2 (2.1 to 2.3)	44.4	<0.001	2.9 (2.9 to 3.0)	61.1	<0.001	1.4 (1.2 to 1.5)	16.1	<0.001	1.9 (1.8 to 2.0)	31.1	<0.001
	2000–2009	4.3 (4.1 to 4.5)	50.5	<0.001	1.6 (1.4 to 1.7)	25.8	<0.001	0.3 (−0.2 to 0.7)	1.1	0.28	0.7 (0.6 to 0.9)	93	<0.001
	2010–2021	1.6 (1.42 to 1.7)	22.0	<0.001	1.0 (0.9 to 1.1)	17.1	<0.001	−0.1(−0.2 to 0.1)	−0.9	0.37	0.3 (0.2 to 0.5)	4.4	<0.001
	1990–2021	0.9 (0.72 to 1.1)	99	<0.001	1.8 (1.7 to 1.9)	52.5	<0.001	0.5 (0.3 to 0.7)	6.3	<0.001	1.0 (0.9 to 1.0)	21.9	<0.001

AAPC, average annual percentage change; DALYs, disability-adjusted life-years.

**Table 2:** The incidence and DALYs of Parkinson’s disease AAPCs from 1990 to 2021 at the global and regional levels.

	Incidence							DALYs						
	Cases (n), 1990	Incidence (per 100000 population), 1990	Cases (n), 2021	Incidence (per 100000 population), 2021	AAPC, 1990–2021	*t*	*p* value	Cases (n), 1990	DALYs (per 100000 population), 1990	Cases (n), 2019	DALYs (per 100000 population), 2021	AAPC, 1990–2021	*t*	*p* value
Global	28 267 (37 341–20 674)	1.0 (1.4–0.8)	81 047 (109 007–59 128)	2.1 (2.8–1.5)	0.9 (0.72 to 1.1)	52.5	<0.001	110 204 (78 590–110 204)	3.4 (4.1–2.9)	216 696 (151 051–216 696)	4.6 (5.5–3.8)	1.0 (0.9 to 1.0)		<0.001
Sex														
Male	16 438 (21 634–12 080)	1.2 (1.6–0.9)	48 417 (64 905–35 786)	2.4 (3.2–1.8)	2.3 (2.2 to 2.4)	53.7	<0.001	56 869 (67 545–47 822)	4.1 (4.9–3.5)	114 178 (136 858–95 125)	5.7 (6.8–4.8)	1.1 (1.0 to 1.2)		<0.001
Female	11 828 (15 577–8 474)	0.9 (1.2–0.6)	32 629 (43 843–23 308)	1.7 (2.2–1.2)	0.8 (0.6 to 1.1)	45.3	<0.001	35 706 (43 808–29 368)	2.7 (3.3–2.2)	66 147 (82 192–54 338)	3.4 (4.2–2.8)	0.8 (0.7 to 0.9)		<0.001
Age group, years														
15–20	NA	NA	NA	NA	NA	NA	NA	NA	NA	NA	NA	NA	NA	NA
20–24	618 (1 206–159)	0.1 (0.2–0.0)	812 (1 584–203)	0.1 (0.3–0.0)	0.3 (0.2 to 0.3)	12.1	<0.001	1 210 (1 450–1 007)	0.2 (0.3–0.2)	1 184 (1 427–993)	0.2 (0.3–0.2)	−0.7 (−0.8 to –0.6)	−13.9	<0.001
25–29	1 670 (3 262–432)	0.4 (0.7–0.1)	2383 (4630–595)	0.4 (0.8–0.1)	0.2 (0.2 to 0.3)	13.8	<0.001	2 224 (3 290–1 411)	0.5 (0.7–0.3)	2 905 (4 410–1 757)	0.5 (0.7–0.3)	−0.05 (−0.15 to 0.05)	−1.01	0.31
30–34	3 023 (4 429–1 849)	0.8 (1.1–0.5)	5 516 (8 030–3 415)	0.9 (1.3–0.6)	0.5 (0.5 to 0.5)	47.3	<0.001	4 535 (6 719–2 897)	1.2 (1.7–0.8)	7 158 (10 755–4 495)	1.2 (1.8–0.7)	0.03 (−0.07 to 0.13)	0.53	0.60
35–39	4 715 (8 703–1 909)	1.3 (2.5–0.5)	9 324 (16 564–3 736)	1.7 (3.0–0.7)	0.7 (0.7 to 0.7)	41.5	<0.001	8 000 (11 285–5 416)	2.3 (3.2–1.5)	13 700 (19 941–8 976)	2.4 (3.6–1.6)	0.23 (0.2 to 0.3)	7.67	<0.001
40–44	7 516 (10 682–5 026)	2.6 (3.7–1.8)	20 478 (28 054–14 231)	4.1 (5.6–2.8)	1.4 (1.3 to 1.5)	23.5	<0.001	29 663 (35 251–24 954)	10.4 (12.3–8.7)	52 905 (64 619–43 535)	10.6 (12.9–8.7)	0.07 (−0.03 to 0.2)	1.41	0.16
45–49	10 724 (16 851–5 779)	4.6 (7.3–2.5)	42 533 (63 467–25 981)	9 (13.4–5.5)	2.1 (2 to 2.3)	27.7	<0.001	46 944 (54 399–40 755)	20.2 (23.4–17.6)	102 473 (123 879–86 233)	21.6 (26.2–18.2)	0.2 (0.1 to 0.3)	4.06	<0.001
Sociodemographic index														
High-middle	6 058 (8 147–4 317)	1.1 (1.4–0.8)	20 352 (27 880–14 327)	3.2 (4.4–2.3)	3.6 (3.5 to 3.7)	59.6	<0.001	20 402 (24 032–17 227)	3.6 (4.3–3.1)	35 211 (44 792–28 717)	5.6 (7.1–4.6)	1.4 (1.3 to 1.6)	17.8	<0.001
High	5 453 (7 326–3 841)	1.2 (1.6–0.8)	8 406 (11 154–6 168)	1.7 (2.2–1.2)	1.1 (1.1 to 1.2)	23.1	<0.001	15 176 (18 473–12 827)	3.3 (4.0–2.8)	20 989 (24 905–17 914)	4.2 (5.0–3.6)	0.8 (0.6 to 1.0)	7.2	<0.001
Low-middle	5 527 (7 261–4 040)	1.0 (1.3–0.7)	14 599 (19 209–10 690)	1.4 (1.9–1.1)	1.1 (1.1 to 1.2)	49.2	<0.001	17 112 (20 809–14 197)	3.1 (3.8–2.6)	40 011 (48 097–33 094)	3.9 (4.7–3.3)	0.8 (0.7 to 0.8)	25.5	<0.001
Low	1 830 (2 440–1 328)	0.8 (1.1–0.6)	5 073 (6 723–3 693)	0.9 (1.2–0.7)	0.4 (0.3 to 0.4)	10.7	<0.001	6 472 (7 920–5 283)	2.9 (3.6–2.4)	16 030 (19 682–12 825)	3.0 (3.6–2.4)	0.02 (−0.09 to 0.13)	0.4	0.69
Middle	9 373 (12 239–6 916)	1.0 (1.3–0.8)	32 574 (43 741–23 784)	2.6 (3.5–1.9)	3.0 (2.9 to 3.2)	39.2	<0.001	33 337 (39 181–28 259)	3.7 (4.3–3.1)	67 961 (81 772–56 412)	5.4 (6.5–4.5)	1.3 (1.2 to 1.4)	24.5	<0.001
Region														
East Asia	7 403 (9 756–5 436)	1.1 (1.4–0.8)	34 920 (47 959–24 299)	5.1 (7–3.5)	5.1 (4.8 to 5.5)	33.9	<0.001	30 864 (36 346–25 543)	4.5 (5.3–3.7)	53 089 (68 103–42 492)	7.7 (9.9–6.2)	1.8 (1.7 to 1.9)	28.2	<0.001
Oceania	26 (35–18)	0.8 (1.1–0.6)	73 (99–50)	1.0 (1.4–0.7)	0.8 (0.7 to 0.9)	24.1	<0.001	85 (110–64)	2.7 (3.4–2.0)	208 (269–160)	2.9 (3.8–2.3)	0.3 (0.3 to 0.4)	11.7	<0.001
Central Asia	226 (325–150)	0.7 (1.0–0.5)	442 (618–299)	0.9 (1.3–0.6)	0.9 (0.9 to 1)	26.6	<0.001	709 (878–589)	2.1 (2.6–1.8)	1 306 (1 615–1 069)	2.7 (3.3–2.2)	0.7 (0.4 to 1.1)	4.4	<0.001
Central Europe	564 (786–379)	0.9 (1.3–0.6)	576 (811–388)	1.1 (1.5–0.7)	0.6 (0.5 to 0.7)	18.1	<0.001	1830 (2 159–1 572)	2.9 (3.5–2.5)	1 666 (1 958–1 421)	3.2 (3.7–2.7)	0.2 (0 to 0.4)	2.3	0.02
Eastern Europe	1 237 (1 694–852)	1.1 (1.5–0.8)	1 421 (1 955–957)	1.5 (2.0–1.0)	0.9 (0.8 to 1.1)	13.6	<0.001	2780 (3493–2246)	2.5 (3.2–2.0)	3 486 (4 373–2 870)	3.6 (4.5–3.0)	1.2 (0.7 to 1.7)	4.7	<0.001
High-income Asia Pacific	898 (1227–605)	1.0 (1.3–0.7)	1 036 (1 468–700)	1.3 (1.9–0.9)	1.0 (0.9 to 1.1)	14.2	<0.001	2 816 (3 371–2 380)	3.0 (3.6–2.6)	3058 (3 697–2 538)	3.9 (4.7–3.2)	0.8 (0.6 to 1.1)	6.4	<0.001
Australasia	54 (77–32)	0.5 (0.7–0.3)	106 (158–66)	0.7 (1.1–0.5)	1.3 (1.2 to 1.4)	26.6	<0.001	201 (248–170)	1.9 (2.3–1.6)	374 (461–314)	2.6 (3.2–2.2)	1.1 (0.9 to 1.2)	14.2	<0.001
Western Europe	2 787 (3 753–1 923)	1.4 (1.9–1.0)	3 597 (4 933–2 413)	1.9 (2.6–1.3)	0.9 (0.9 to 1)	23.9	<0.001	6 379 (8 262–5 044)	3.3 (4.3–2.6)	7 347 (9 565–5 654)	3.9 (5.1–3)	0.5 (0.4 to 0.7)	6.9	<0.001
Southern Latin America	163 (234–96)	0.7 (1.0–0.4)	327 (465–201)	0.9 (1.3–0.6)	1.2 (1 to 1.3)	19	<0.001	567 (677–480)	2.3 (2.8–2.0)	878 (1 095–719)	2.5 (3.2–2.1)	0.3 (0.1 to 0.5)	2.9	<0.001
High-income North America	1 760 (2 327–1 276)	1.2 (1.6–0.9)	1 921 (2 357–1 558)	1.1 (1.4–0.9)	−0.1 (−0.2 to 0)	−1.4	0.16	4 877 (5 934–4 074)	3.3 (4.0–2.7)	6 195 (6 923–5 553)	3.7 (4.1–3.3)	0.4 (0.2 to 0.6)	4	<0.001
Caribbean	228 (305–167)	1.2 (1.7–0.9)	434 (578–315)	1.8 (2.4–1.3)	1.2 (1.1 to 1.3)	26.3	<0.001	608 (744–488)	3.3 (4.1–2.7)	1 141 (1 403–911)	4.8 (5.9–3.8)	1.2 (1 to 1.3)	15.3	<0.001
Andean Latin America	412 (527–310)	2.2 (2.8–1.7)	1 429 (1 860–1 061)	4.1 (5.3–3)	2.0 (1.9 to 2.1)	104.2	<0.001	797 (1 049–616)	4.3 (5.6–3.3)	2 052 (2 770–1 539)	5.9 (7.9–4.4)	1.0 (0.8 to 1.2)	10.9	<0.001
Central Latin America	1 023 (1 337–749)	1.3 (1.6–0.9)	2 819 (3 702–2 087)	2.1 (2.8–1.6)	1.7 (1.6 to 1.8)	39.8	<0.001	2 806 (3 393–2 342)	3.4 (4.2–2.9)	6 730 (8 408–5 502)	5.1 (6.3–4.1)	1.3 (1.2 to 1.4)	27.4	<0.001
Tropical Latin America	1 002 (1 310–738)	1.3 (1.7–0.9)	2 648 (3 536–1 930)	2.2 (3–1.6)	1.8 (1.7 to 1.9)	36.2	<0.001	2 282 (2 829–1 846)	2.9 (3.6–2.4)	5 525 (7 046–4 408)	4.6 (5.9–3.7)	1.5 (1.4 to 1.5)	51.4	<0.001
North Africa and Middle East	1 354 (1 785–984)	0.8 (1.1–0.6)	5 122 (6 749–3 725)	1.5 (2–1.1)	1.9 (1.9 to 2)	66.6	<0.001	5 237 (6 292–4 305)	3.3 (3.9–2.7)	14 679 (17 826–12 167)	4.4 (5.3–3.6)	1.0 (0.9 to 1.1)	24.7	<0.001
South Asia	5 583 (7 386–4 071)	1.1 (1.4–0.8)	15 225 (20 372–11 082)	1.5 (2–1.1)	1.1 (1 to 1.2)	33.4	<0.001	15 852 (19 605–12 827)	3.0 (3.7–2.4)	38 479 (47 689–30 653)	3.8 (4.7–3)	0.8 (0.7 to 0.9)	15.5	<0.001
Central sub- Saharan Africa	172 (237–119)	0.7 (1–0.5)	543 (743–373)	0.8 (1.1–0.6)	0.5 (0.5 to 0.6)	34.3	<0.001	588 (752–442)	2.4 (3.1–1.8)	1 695 (2 173–1 281)	2.6 (3.3–2)	0.3 (0.1 to 0.4)	4.1	<0.001
Eastern sub- Saharan Africa	605 (812–440)	0.7 (1.0–0.5)	1 647 (2 210–1 181)	0.8 (1.1–0.6)	0.3 (0.2 to 0.3)	15.3	<0.001	2 004 (2 447–1 592)	2.4 (2.9–1.9)	4 885 (6 256–3 782)	2.3 (3–1.8)	−0.1 (–0.1 to 0)	−3.2	<0.001
Southern sub- Saharan Africa	197 (263–145)	0.8 (1–0.6)	434 (575–316)	1.0 (1.3–0.7)	0.9 (0.8 to 0.9)	37.5	<0.001	714 (836–605)	2.8 (3.2–2.4)	1 737 (2 046–1 459)	4.0 (4.7–3.4)	1.1 (1 to 1.3)	12.4	<0.001
Western sub- Saharan Africa	547 (746–384)	0.6 (0.9–0.4)	1 620 (2 188–1 129)	0.7 (1–0.5)	0.3 (0.3 to 0.4)	15.9	<0.001	2 583 (3 155–2 089)	3.0 (3.7–2.4)	7 339 (10 419–5 426)	3.2 (4.5–2.4)	0.2 (0.1 to 0.3)	3.6	<0.001
Southeast Asia	2 026 (2 713–1 478)	0.9 (1.1–0.6)	4 706 (6 425–3 389)	1.3 (1.7–0.9)	1.3 (1.2 to 1.4)	48.4	<0.001	7 996 (9489–6849)	3.4 (4.0–2.9)	18 458 (22 027–15 751)	5.0 (5.9–4.2)	1.3 (1.2 to 1.3)	30.5	<0.001

Data in parentheses are 95% uncertainty intervals for cases, incidence, and DALYs, and 95% CIs for AAPCs. DALYs, disability-adjusted life-years; UI, uncertainty interval; NA, not available; AAPC, average annual percentage change.

Globally, there was an increase in the prevalence of EOPD in both men and women from 1990 to 2021, with a 2.3 AAPC (95% CI 0.72 to 1.1; rising from 1.2 per 100,000 population [95% UI 1.6–0.9] in 1990 to 2.4 per 100,000 population [3.2–1.8] in 2021) in men and 0.8 (95% CI 0.6 to 1.1; increasing from 0.9 per 100,000 population [95% UI 1.2–0.6] to 1.7 per 100,000 population [2.2–1.2]) in women (see Table 2). Globally, 81,047 cases of EOPD were reported in 2021, with 48,417 (59.7%) occurrences in males and 32,629 (40.3%) occurrences in females (Table 2). Both men and women experienced an increase in DALYs from 1990 to 2021, with a 1.1 AAPC for males (95% CI 1.0 to 1.2; rising from 4.1 per 100,000 population [95% UI 4.9–3.5] in 1990 to 5.7 per 100,000 population [6.8–4.8] in 2021) and a 0.8 AAPC for females (95% CI 0.7 to 0.9; increasing from 2.7 per 100,000 population [95% UI 3.3–2.2] to 3.4 per 100,000 population [4.2–2.8]) as shown in Table 2.

10,724 (37.1%) of the 28,267 cases of EOPD reported in 2021 were among individuals aged 45–49. The most substantial increase in EOPD cases from 1990 to 2021 was observed in individuals aged 45–49 years, with a rise from 4.6 per 100,000 people [95% UI 7.3–2.5] in 1990 to 9 per 100,000 people [13.4–5.5] in 2021. The AAPC was 2.1 [95% CI 2 to 2.3]. The incidence of EOPD was also increasing among individuals aged 20–44 years; however, there was no data available on the incidence of EOPD among adolescents aged 15–20 years. Between 1990 and 2019, there was an increase in EOPD DALYs in the 30–49 age bracket, with the largest rise seen in the 45–49 age range (AAPC 0.2 [95% CI 0.1 to 0.3]) (rising from 20.2 per 100,000 population [95% UI 23.4–17.6] in 1990 to 21.6 per 100,000 population [26.2–18.2] in 2021). In contrast, there was a reduction in DALYs among individuals aged 20–24 years (AAPC −0.7 [95% CI −0.8 to −0.6]) and those aged 25–29 years (AAPC-0.05 [95% CI −0.15 to 0.05]) according to Table 2.

The incidence of EOPD has increased in all five SDI regions since 1990. In 2021, the high-middle SDI region saw a substantial rise in EOPD cases, reaching a rate of 3.2 per 100,000 people [95% UI 4.4 to 2.3], compared to 1.1 per 100,000 people [1.4 to 0.8] in 1990 (AAPC 3.6 [95% CI 3.5 to 3.7]). The low SDI area saw minimal growth in EOPD incidence, rising from 0.8 per 100,000 people (95% UI 1.1 to 0.6) in 1990 to 0.9 per 100,000 individuals (95% UI 1.2 to 0.7) in 2021, with an AAPC of 0.4 (95% CI 0.3 to 0.4) (Table 2).

The largest increases in EOPD rates from 1990 to 2021 were seen in East Asia, Andean Latin America, and North Africa and the Middle East. EOPD rates have only declined in affluent North America, dropping from 1.2 to 1.1 per 100,000 people, according to Table 2 (AAPC-0.1 [95% CI −0.2–0]).

Between 1990 and 2021, EOPD caused the largest rise in DALYs in East Asia, with rates increasing from 4.5 to 7.7 per 100,000 people (95% UI 5.3 to 3.7); AAPC 1.8 (95% CI 1.7 to 1.9). Eastern sub-Saharan Africa is the sole region globally that saw a reduction in DALYs from EOPD, dropping from 2.4 per 100,000 population to 2.3 per 100,000 population between 1990 and 2021, with an AAPC of −0.1.

**Figure 2:**
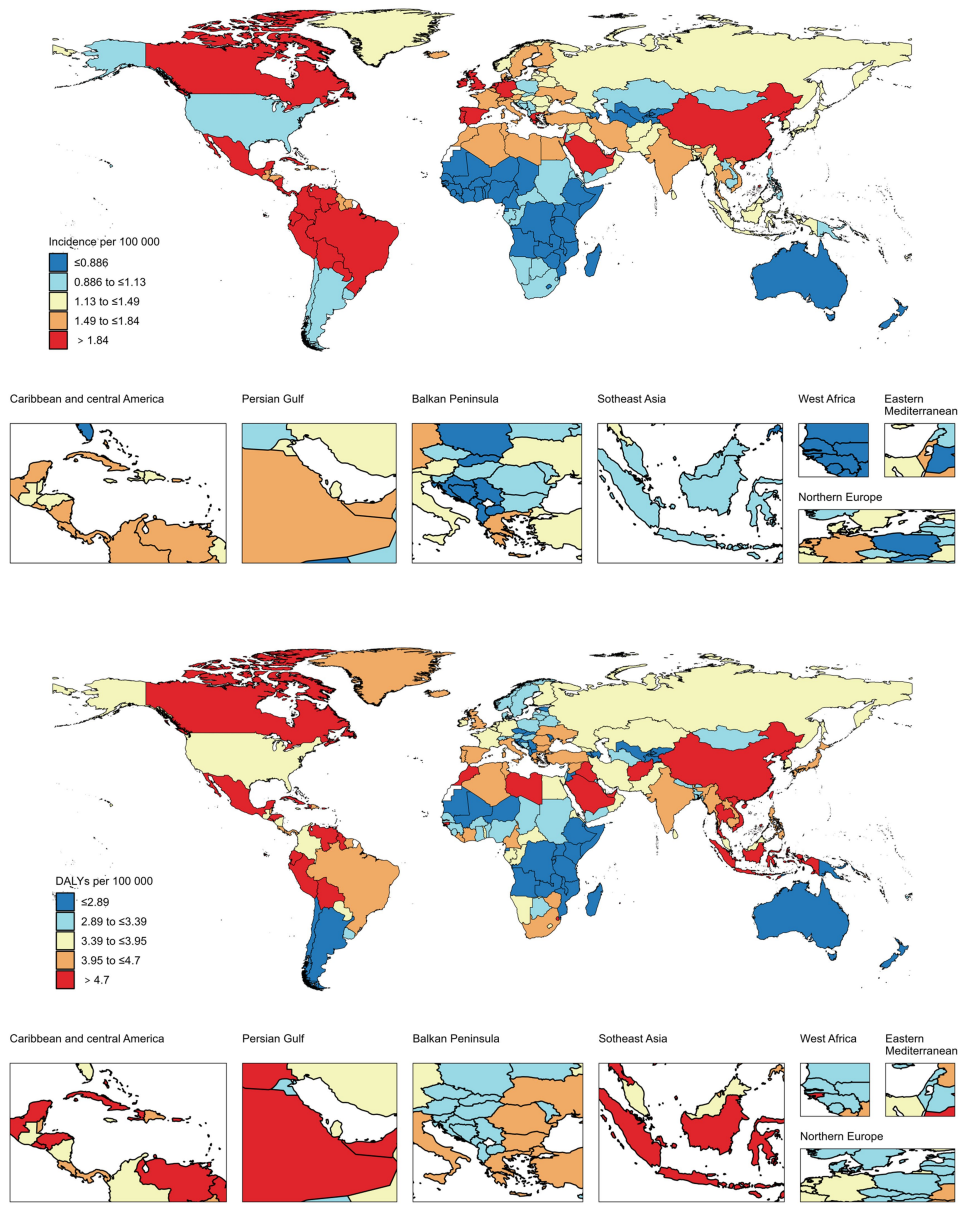
Global EOPD incidence and DALYs within individual GBD regions.

China experienced the highest rate of EOPD in 2021, with a incidence of 5.17 per 100,000 people (95% UI 7.12–3.59) (see Figure 2 and Supplementary Table 1). Following that is Peru with a rate of 4.23 per 100,000 individuals [95% UI 5.58–3.04], followed by Bolivia (Plurinational State of) with a rate of 3.92 per 100,000 people [95% UI 5.22–2.90]. Saudi Arabia recorded the highest number of DALYs from EOPD in 2021, with a rate of 8.97 per 100,000 people and a 95% UI of 11.58–6.83. The countries with the highest suicide rates are North Korea (8.75 per 100,000 people [95% UI 11.98–6.13]) and Seychelles (8.07 per 100,000 people [95% UI 9.74–6.80]) (Supplementary Table 1).

### Decomposition analysis of incidence and DALYs in EOPD population

**Figure 3:**
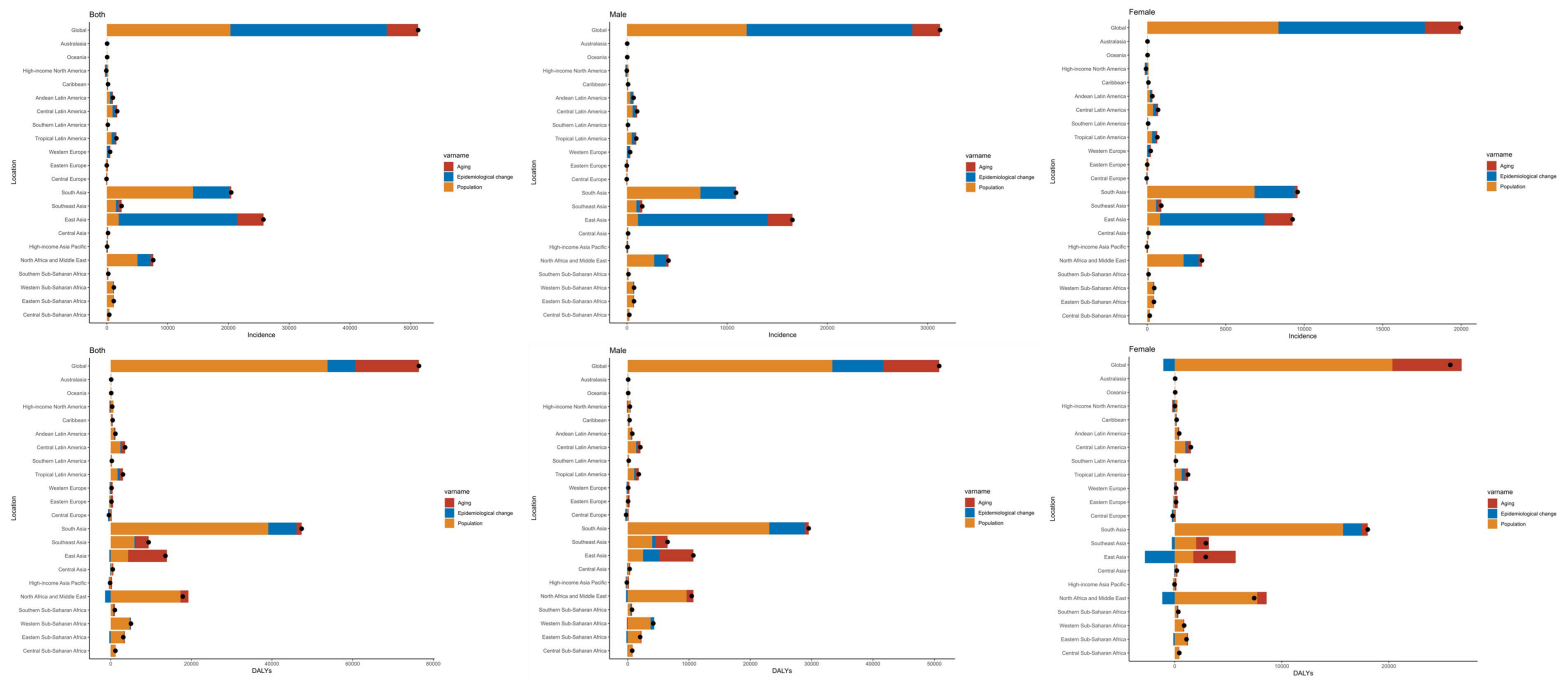
Decomposition analysis of incidence and DALYs in EOPD population in GBD regions.

We observed a significant increase in the global EOPD incidence within individual GBD regions, with the largest increase occurring in East Asia between 1990 and 2021 (Figure 3). Our results indicate that the burden of EOPD has increased by 50.31 and 39.71%, respectively, between 1990 and 2021, as a result of epidemiological changes and population growth (Supplementary Table 2). There were comparable patterns observed among both genders. In the GBD regions, the incidence of EOPD increased most significantly in East Asia, South Asia, and North Africa and the Middle East. The primary cause of the change in East Asia was epidemiological changes (75.76%), while population growth (69.58 and 69.27%, respectively) was the primary cause of the change in South Asia, North Africa, and the Middle East. The primary cause of high-income North America was population growth (−241.73%), while aging was the primary cause of Central Europe and Eastern Europe (−101.43% and −770.13%, respectively) (Figure 3 and Supplementary Table 2).

According to a decomposition analysis, the burden of DALYs increased by 70.37% between 1990 and 2021 as a result of population growth. Men were more responsible for the increase in the burden of DALYs than women, and the decomposition analysis was consistent with the global findings. Nevertheless, the female population experienced a negative impact due to epidemiological changes (−4.24%) (Figure 3 and Supplementary Table 3).

Supplementary Table 3 provides an analysis of the decomposition of the DALYs number variations in the GBD regions between 1990 and 2021. In terms of GBD regions, the decomposition of EOPD DALYs indicated that the primary cause of the increase was population growth, with the highest burden being experienced in South Asia, North Africa, the Middle East, and East Asia. The Middle East, North Africa, and South Asia were predominantly impacted by population growth, while East Asia was primarily impacted by aging.

## Discussion

This study addresses a substantial gap in our comprehension of the epidemiology of EOPD by offering the first comprehensive global analysis of its trends from 1990 to 2021. The incidence of EOPD worldwide increased from 1990 to 2021, with a particularly rapid increase between 2000 and 2009. The 45–49 age group exhibited the most significant increase in EOPD incidence and DALYs, with males having a higher incidence than females. In 2021, China had the highest country-specific incidence of EOPD, and East Asia experienced the greatest regional increase in DALYs and EOPD incidence. The global burden of EOPD has been significantly influenced by population growth and epidemiological changes.

It is crucial to comprehend the observed global increase in EOPD incidence in the context of this background. The primary factors contributing to the global increase in EOPD incidence are likely to be enhanced diagnosis and awareness (21). The improved identification and reporting of EOPD cases have been facilitated by the increased recognition and awareness of EOPD among healthcare professionals and the public, as well as technological advancements in diagnostic techniques such as neuroimaging and genetic testing (4, 22, 23). Furthermore, the advent of Parkinson’s disease may have been influenced by globally varying lifestyle trends and dietary patterns during this time. Prior studies indicate a connection between the Mediterranean-DASH Intervention for Neurodegenerative Delay (MIND) and Mediterranean diets with the development of Parkinson’s disease in older age (24, 25). Additionally, the risk of developing Parkinson’s disease, including the early-onset form, may rise as the global population ages. It is important to note that the increase in the incidence of EOPD may be influenced by the aging of the global population during this period (26).

Various factors, such as biological, environmental, lifestyle, and socioeconomic factors, contribute to the gender gap. Our research indicates that males have a higher incidence of EOPD and DALY rates than females, which is in line with previous research (27–29). In their study, Popat and Nelson (30) investigate the impact of reproductive factors on the age at which Parkinson’s disease manifests, as well as the sex differences that exist. Furthermore, it investigates the influence of biological factors, including estrogen levels, on the progression of the disease. Previous research has shown that Parkinson’s disease is more common in men than in women, with higher rates of both occurrence and development (27, 28).

Research indicates that the frequency of Parkinson’s disease (PD) in East Asian areas is often less than in Europe and North America (31). The incidence of EOPD and DALYs in East Asia, however, exhibited the most significant increase in our study. PD has experienced a substantial increase in recent years in a number of Asian countries, such as China, South Korea, Japan, Thailand, and Israel, necessitating increased care and attention (32). The prevalence of Parkinson’s disease (PD) in East Asian regions is influenced by a variety of factors, including age, gender, environmental exposures, genetic predispositions, and socioeconomic determinants, according to emerging research. It is important to note that the characteristics of PD in East Asia seem to deviate from the patterns observed in Europe and North America (31, 33, 34). Despite the limited research on early-onset Parkinson’s disease (EOPD) in China, some studies have hinted that the prevalence of EOPD is on the rise (35–39). According to a published study, there is a current increase in the prevalence of EOPD, which may be associated with PD as well (40). For example, a recent projection analysis conducted by Chen et al. (37), demonstrated that the projected age-specific PD incidence for both genders is on the increase from 2020 to 2030. This implies that the burden of PD for all age groups and both genders in China may increase. In addition, it was determined that the risk of PD mortality was lower in younger generations (those born in later birth cohorts), while the incidence risk increased. PD was discovered to be associated with lifestyle factors, socioeconomic status, education, and other factors (37, 41). Partly as a result of improved living conditions and partly due to improved education, younger generations in China tend to live healthier and be more health-conscious than older generations (37, 42).

The worldwide increase in the burden of EOPD can be primarily attributed to the significant epidemiological shifts and population growth dynamics that have occurred. The increased prevalence and impact of this debilitating neurological disorder have been significantly influenced by these interrelated factors. The number of individuals at risk of developing Parkinson’s disease, including the early-onset form, has increased globally as a result of the increase in life expectancy (26). T The likelihood of developing Parkinson’s disease increases with age, leading to a rise in early-onset Parkinson’s disease cases as the population ages. This aligns with our research findings, as the age group of 45–49 years has the maximum incidence rate of EOPD and is also the oldest group in the EOPD age range. The increasing burden of EOPD has been influenced by changes in diagnostic criteria and clinical practices, in addition to the aging population. In recent decades, there have been substantial improvements in the comprehension of Parkinson’s disease, resulting in more precise and sensitive diagnostic instruments. This has made it easier to identify and report cases of EOPD that were previously classified incorrectly or not diagnosed properly. A variety of innovative methods, such as wearable technologies, automated neuromelanin imaging, machine learning methodologies, and the integrated analysis of novel biomarkers, are employed in the development of new diagnostic instruments for PD (43–47). Additionally, the incidence of EOPD has been linked to environmental and lifestyle factors that are associated with accelerated urbanization and industrialization. The development of Parkinson’s disease at a younger age has been associated with sedentary lifestyles, poor dietary practices, and exposure to environmental toxins, such as pesticides and air pollution (48–51). In the context of the global epidemiological transition, where developing countries are undergoing rapid socioeconomic changes, these modifiable risk factors are particularly relevant. Finally, the increasing burden of EOPD has been exacerbated by the rapid expansion of the global population, particularly in developing regions (52, 53). The prevalence and impact of EOPD have increased as a result of the increasing number of individuals at risk, which is a direct result of the global population’s continued growth. Studying changes in disease patterns and population characteristics can lead to a better understanding of the reasons behind the rising prevalence of early-onset Parkinson’s disease worldwide.

The GBD data plays a crucial role in informing our findings on EOPD, particularly regarding trends in incidence, prevalence, and DALYs. Our analysis revealed a significant increase in the global incidence of EOPD from 1990 to 2021, with a notable rise during the period from 2000 to 2009, where the AAPC was 4.3%. This trend highlights the importance of enhanced diagnosis and awareness, as improved identification of EOPD cases has been facilitated by advancements in diagnostic techniques and increased recognition among healthcare professionals (18). To enhance the quality and reliability of data collection on EOPD, several improvements are necessary. First, reporting practices in low-and middle-income countries should be standardized to ensure consistency in data collection and reporting. This could involve the establishment of national registries for EOPD that adhere to uniform definitions and diagnostic criteria, thereby facilitating accurate data capture (17). Additionally, increasing the granularity of data collection—such as stratifying data by age, sex, and socioeconomic status—would provide deeper insights into the epidemiological trends and risk factors associated with EOPD. Lastly, fostering collaboration among researchers, healthcare providers, and policymakers can help address the gaps in data coverage and improve the overall understanding of EOPD, ultimately guiding targeted public health interventions and resource allocation.

The increasing global burden of EOPD, particularly in specific regions and age groups, emphasizes the imperative necessity for strategic resource allocation and tailored interventions in healthcare. In order to confront the distinctive obstacles of EOPD, it is imperative to implement proactive public health measures in this changing environment. Healthcare decision-makers must prioritize early detection and specialized EOPD care. In order to mitigate the profound impact on patients and families, it is essential to implement comprehensive strategies, such as personalized treatment, expeditious diagnosis, and multidisciplinary support. The quality of care can be improved by allocating resources to EOPD clinics, expanding access to novel diagnostics, and training professionals. In order to enhance comprehension of the underlying causes and risk profiles of EOPD, it is imperative to increase research funding and foster cross-disciplinary collaboration. Targeted prevention and intervention strategies will be informed by this. Exploring innovative therapies has the potential to transform the management of EOPD and enhance long-term results. The profound individual and societal impacts of this challenging neurological disorder can be mitigated through a multifaceted approach that incorporates clinical best practices and public health initiatives.

This work’s primary strength is its exhaustive examination of global EOPD trends, which is based on comprehensive data from the GBD 2021 database, although it is important to note that the data quality and availability vary significantly across different regions, particularly in low-and middle-income countries. The study offers a comprehensive comprehension of this evolving public health challenge by analyzing the temporal and spatial dynamics, as well as decomposing incidence and DALY changes. Nevertheless, our investigation was subject to numerous constraints. While the GBD data provides valuable insights into EOPD trends, our investigation is limited by the varying quality of this data and significant gaps in coverage, particularly from low-and middle-income countries, which may affect the overall interpretation of the findings. Additionally, we believe there is a moderate likelihood that EOPD is not accurately reported and recognized worldwide, which may significantly impact the validity of our results. This assessment is based on the observed discrepancies in data reporting practices across different regions and the challenges in diagnosing EOPD. The GBD database was the only data source used in this study, unlike other global databases like the WHO’s Global Health Estimates, which compile data from multiple sources. Moreover, there is a risk of inaccuracies and prejudices being introduced due to the dependence on epidemiological models and data reporting methods, potentially leading to an underestimation of the actual impact of EOPD. The capacity to develop targeted interventions and preventive strategies is further restricted by the absence of detailed information on the underlying causes and risk factors of EOPD. Enhancing data quality, studying specific populations such as adolescents, utilizing diverse data sources beyond GBD for accuracy, exploring gender intersectionality, conducting longitudinal studies to track trends, and collaborating to develop targeted prevention strategies that address root causes and risks are potential areas of future research on EOPD. In summary, this investigation provides a valuable insight into the global burden of EOPD, while simultaneously emphasizing the necessity of additional research to address the knowledge voids and fortify the evidence base to facilitate effective policymaking.

## Conclusion

This comprehensive global analysis of EOPD trends from 1990 to 2021 offers critical insights into the changing epidemiology of this debilitating neurological disorder. The significant need for focused public health actions and research agendas is highlighted by the documented rise in EOPD cases worldwide, especially the rapid expansion from 2000 to 2009. The significance of comprehending gender-specific and age-related risk factors is underscored by the disproportionate impact on the 45–49 age group and the higher EOPD burden among males compared to females. Customized strategies are required to address the distinct challenges faced by populations in East Asia, Andean Latin America, and North Africa/Middle East due to the substantial regional disparities and significant increases experienced in these areas. A valuable framework for the development of evidence-based policies and resource allocation is provided by the decomposition analysis, which identified epidemiological changes and population growth as the primary drivers of the increasing EOPD burden. Future research should concentrate on the following: the improvement of data quality, the investigation of the underlying causes and risk factors, and the implementation of longitudinal studies to more effectively monitor the changing trends. To strengthen the evidence foundation, additional data sources can be integrated apart from the Global Burden of Disease Study. The profound individual and societal impacts of this challenging neurological disorder necessitate collaborative efforts among clinicians, researchers, and public health professionals. Early detection, personalized treatment, and multidisciplinary support are proactive measures that can alleviate the burden of EOPD and enhance long-term outcomes for patients and their families worldwide.

## Data Availability

The original contributions presented in the study are included in the article/Supplementary material, further inquiries can be directed to the corresponding author.
