# Supplementary material for: Temporal and geographical dynamics of early-onset Parkinson’s disease burden: insights from the Global Burden of Disease Study 2021
Source: Front Neurol. 2025 Jan 30;16:1473548. doi: 10.3389/fneur.2025.1473548 (PMC11821659; doi:10.3389/fneur.2025.1473548)
Supplement: Supplementary file 1 [file Table_1.DOCX]

Supplementary Table 1 Global EOPD incidence and DALYs in individual countries

| Locations | Incidence (per 100000 population) | Incidence 95% UI upper | incidence 95% UI lower | DALYs(per 100000 population) | DALYs 95% UI upper | DALYs 95% UI lower |
| --- | --- | --- | --- | --- | --- | --- |
| Afghanistan | 1.27 | 1.68 | 0.96 | 6.06 | 9.50 | 3.40 |
| Albania | 0.94 | 1.36 | 0.57 | 2.34 | 3.11 | 1.78 |
| Algeria | 1.51 | 2.05 | 1.05 | 4.08 | 5.20 | 3.17 |
| American Samoa | 1.08 | 1.53 | 0.66 | 3.64 | 4.60 | 2.81 |
| Andorra | 2.19 | 3.27 | 1.35 | 4.12 | 5.65 | 2.83 |
| Angola | 0.83 | 1.14 | 0.57 | 2.62 | 3.37 | 1.98 |
| Antigua and Barbuda | 2.07 | 2.98 | 1.44 | 4.38 | 5.78 | 3.28 |
| Argentina | 0.93 | 1.33 | 0.57 | 2.46 | 3.12 | 1.98 |
| Armenia | 0.95 | 1.34 | 0.59 | 2.22 | 2.92 | 1.70 |
| Australia | 0.75 | 1.14 | 0.45 | 2.55 | 3.21 | 2.11 |
| Austria | 1.75 | 2.56 | 1.14 | 3.36 | 4.60 | 2.40 |
| Azerbaijan | 0.87 | 1.24 | 0.57 | 2.24 | 2.97 | 1.67 |
| Bahamas | 2.13 | 2.92 | 1.49 | 6.43 | 8.08 | 4.91 |
| Bahrain | 1.53 | 2.19 | 1.00 | 3.59 | 4.81 | 2.72 |
| Bangladesh | 1.28 | 1.76 | 0.87 | 2.98 | 4.13 | 2.06 |
| Barbados | 2.13 | 2.86 | 1.48 | 4.79 | 6.40 | 3.55 |
| Belarus | 1.34 | 1.99 | 0.81 | 3.21 | 4.21 | 2.49 |
| Belgium | 1.83 | 2.65 | 1.17 | 3.71 | 5.08 | 2.76 |
| Belize | 1.70 | 2.23 | 1.24 | 4.46 | 5.59 | 3.59 |
| Benin | 0.70 | 0.98 | 0.48 | 3.11 | 4.40 | 2.18 |
| Bermuda | 2.60 | 3.59 | 1.78 | 4.99 | 7.09 | 3.41 |
| Bhutan | 1.33 | 1.87 | 0.89 | 3.38 | 4.48 | 2.49 |
| Bolivia (Plurinational State of) | 3.93 | 5.22 | 2.91 | 5.74 | 7.97 | 4.03 |
| Bosnia and Herzegovina | 1.09 | 1.62 | 0.64 | 2.91 | 3.72 | 2.19 |
| Botswana | 1.05 | 1.44 | 0.74 | 3.07 | 4.10 | 2.29 |
| Brazil | 2.22 | 2.97 | 1.61 | 4.65 | 5.93 | 3.71 |
| Brunei Darussalam | 1.22 | 1.68 | 0.83 | 4.45 | 5.75 | 3.05 |
| Bulgaria | 1.15 | 1.64 | 0.70 | 4.61 | 5.50 | 3.89 |
| Burkina Faso | 0.68 | 0.94 | 0.46 | 3.09 | 4.20 | 2.07 |
| Burundi | 0.79 | 1.09 | 0.53 | 2.35 | 3.13 | 1.60 |
| Cabo Verde | 1.02 | 1.43 | 0.71 | 4.73 | 6.31 | 3.57 |
| Cambodia | 1.04 | 1.47 | 0.73 | 4.09 | 5.47 | 3.13 |
| Cameroon | 0.77 | 1.09 | 0.52 | 4.15 | 5.95 | 2.75 |
| Canada | 2.40 | 3.04 | 1.76 | 4.71 | 5.86 | 3.84 |
| Central African Republic | 0.93 | 1.24 | 0.66 | 3.41 | 4.83 | 2.29 |
| Chad | 0.62 | 0.86 | 0.44 | 3.03 | 4.13 | 2.25 |
| Chile | 0.96 | 1.38 | 0.58 | 2.60 | 3.33 | 2.12 |
| China | 5.17 | 7.12 | 3.59 | 7.71 | 9.90 | 6.14 |
| Colombia | 2.00 | 2.73 | 1.39 | 3.95 | 5.27 | 2.98 |
| Comoros | 0.92 | 1.29 | 0.62 | 3.05 | 3.92 | 2.20 |
| Congo | 1.07 | 1.49 | 0.73 | 3.57 | 4.78 | 2.61 |
| Cook Islands | 1.32 | 1.95 | 0.78 | 3.19 | 4.38 | 2.33 |
| Costa Rica | 2.32 | 3.10 | 1.61 | 4.60 | 6.28 | 3.47 |
| Cote d'Ivoire | 0.81 | 1.09 | 0.56 | 4.34 | 6.30 | 3.01 |
| Croatia | 1.05 | 1.61 | 0.60 | 2.56 | 3.28 | 2.03 |
| Cuba | 2.10 | 2.91 | 1.35 | 4.81 | 6.17 | 3.68 |
| Cyprus | 1.69 | 2.42 | 1.07 | 2.96 | 4.16 | 2.06 |
| Czechia | 1.19 | 1.94 | 0.71 | 2.88 | 3.59 | 2.27 |
| Democratic People's Republic of Korea | 2.76 | 3.74 | 1.90 | 8.75 | 11.98 | 6.13 |
| Democratic Republic of the Congo | 0.81 | 1.12 | 0.54 | 2.47 | 3.24 | 1.76 |
| Denmark | 1.79 | 2.60 | 1.12 | 3.16 | 4.56 | 2.20 |
| Djibouti | 0.96 | 1.33 | 0.67 | 2.87 | 4.09 | 2.03 |
| Dominica | 1.81 | 2.43 | 1.29 | 4.99 | 6.67 | 3.80 |
| Dominican Republic | 1.74 | 2.39 | 1.23 | 4.63 | 5.96 | 3.59 |
| Ecuador | 3.89 | 5.14 | 2.81 | 5.50 | 7.79 | 3.88 |
| Egypt | 1.64 | 2.19 | 1.16 | 3.50 | 4.54 | 2.75 |
| El Salvador | 1.90 | 2.69 | 1.32 | 5.38 | 6.82 | 4.11 |
| Equatorial Guinea | 0.78 | 1.09 | 0.55 | 2.32 | 3.20 | 1.66 |
| Eritrea | 0.96 | 1.32 | 0.69 | 3.47 | 4.66 | 2.56 |
| Estonia | 1.54 | 2.24 | 0.97 | 2.68 | 3.72 | 1.95 |
| Eswatini | 1.02 | 1.36 | 0.74 | 4.92 | 6.84 | 3.21 |
| Ethiopia | 0.76 | 1.01 | 0.55 | 1.99 | 2.85 | 1.45 |
| Fiji | 1.20 | 1.62 | 0.80 | 3.28 | 4.27 | 2.57 |
| Finland | 1.83 | 2.64 | 1.17 | 3.61 | 5.11 | 2.62 |
| France | 1.74 | 2.53 | 1.07 | 3.75 | 5.04 | 2.78 |
| Gabon | 1.05 | 1.44 | 0.74 | 3.51 | 4.70 | 2.61 |
| Gambia | 0.69 | 0.98 | 0.47 | 3.22 | 4.88 | 2.14 |
| Georgia | 1.13 | 1.63 | 0.73 | 3.39 | 4.26 | 2.73 |
| Germany | 2.03 | 2.59 | 1.45 | 3.76 | 5.12 | 2.78 |
| Ghana | 0.71 | 0.99 | 0.48 | 3.18 | 4.34 | 2.30 |
| Greece | 1.96 | 2.94 | 1.29 | 4.29 | 5.83 | 3.13 |
| Greenland | 1.13 | 1.50 | 0.78 | 4.26 | 5.39 | 2.99 |
| Grenada | 1.78 | 2.41 | 1.25 | 4.75 | 6.02 | 3.71 |
| Guam | 1.44 | 2.07 | 0.94 | 3.40 | 4.46 | 2.55 |
| Guatemala | 1.55 | 2.10 | 1.09 | 3.80 | 4.86 | 2.97 |
| Guinea | 0.67 | 0.95 | 0.45 | 2.99 | 4.36 | 2.09 |
| Guinea-Bissau | 0.77 | 1.06 | 0.53 | 4.83 | 6.48 | 3.35 |
| Guyana | 1.71 | 2.31 | 1.24 | 5.51 | 7.10 | 4.17 |
| Haiti | 1.55 | 2.11 | 1.14 | 4.72 | 6.28 | 3.28 |
| Honduras | 1.78 | 2.41 | 1.25 | 5.36 | 7.17 | 3.82 |
| Hungary | 1.13 | 1.76 | 0.66 | 3.28 | 3.99 | 2.68 |
| Iceland | 1.83 | 2.63 | 1.18 | 4.02 | 5.30 | 3.04 |
| India | 1.59 | 2.14 | 1.16 | 3.97 | 4.95 | 3.15 |
| Indonesia | 1.20 | 1.63 | 0.87 | 4.79 | 6.04 | 3.83 |
| Iran (Islamic Republic of) | 1.69 | 2.28 | 1.24 | 3.88 | 4.85 | 2.91 |
| Iraq | 1.27 | 1.71 | 0.90 | 4.77 | 6.31 | 3.73 |
| Ireland | 2.01 | 2.94 | 1.26 | 3.58 | 5.07 | 2.53 |
| Israel | 1.93 | 2.76 | 1.25 | 3.19 | 4.54 | 2.26 |
| Italy | 1.79 | 2.48 | 1.21 | 4.46 | 5.86 | 3.46 |
| Jamaica | 1.73 | 2.38 | 1.19 | 3.90 | 5.29 | 2.89 |
| Japan | 1.32 | 1.82 | 0.90 | 3.98 | 4.82 | 3.33 |
| Jordan | 0.94 | 1.31 | 0.66 | 2.74 | 3.45 | 2.15 |
| Kazakhstan | 1.11 | 1.56 | 0.72 | 3.54 | 4.37 | 2.86 |
| Kenya | 0.89 | 1.17 | 0.65 | 2.44 | 3.03 | 1.92 |
| Kiribati | 1.10 | 1.46 | 0.79 | 3.40 | 4.37 | 2.64 |
| Kuwait | 1.55 | 2.22 | 1.05 | 3.31 | 4.38 | 2.48 |
| Kyrgyzstan | 0.78 | 1.12 | 0.49 | 2.35 | 2.99 | 1.84 |
| Lao People's Democratic Republic | 1.02 | 1.40 | 0.70 | 4.07 | 5.17 | 3.13 |
| Latvia | 1.41 | 2.10 | 0.91 | 3.14 | 3.98 | 2.45 |
| Lebanon | 1.28 | 1.79 | 0.91 | 3.05 | 3.95 | 2.36 |
| Lesotho | 0.84 | 1.16 | 0.59 | 3.56 | 4.72 | 2.55 |
| Liberia | 0.79 | 1.10 | 0.51 | 4.02 | 6.50 | 2.60 |
| Libya | 1.70 | 2.28 | 1.20 | 6.58 | 9.71 | 4.67 |
| Lithuania | 1.37 | 2.05 | 0.83 | 3.01 | 4.03 | 2.35 |
| Luxembourg | 1.83 | 2.64 | 1.15 | 3.39 | 4.75 | 2.40 |
| Madagascar | 0.76 | 1.03 | 0.52 | 2.17 | 2.92 | 1.60 |
| Malawi | 0.74 | 1.01 | 0.52 | 2.39 | 3.11 | 1.71 |
| Malaysia | 1.15 | 1.61 | 0.75 | 3.84 | 4.74 | 3.18 |
| Maldives | 1.19 | 1.71 | 0.80 | 2.91 | 4.03 | 2.16 |
| Mali | 0.59 | 0.83 | 0.39 | 2.82 | 4.00 | 2.03 |
| Malta | 1.88 | 2.71 | 1.19 | 4.15 | 5.55 | 3.14 |
| Marshall Islands | 1.35 | 1.83 | 0.95 | 4.65 | 6.00 | 3.45 |
| Mauritania | 0.65 | 0.92 | 0.43 | 2.34 | 3.71 | 1.56 |
| Mauritius | 1.49 | 2.09 | 0.99 | 5.38 | 6.46 | 4.48 |
| Mexico | 2.22 | 2.93 | 1.65 | 5.59 | 6.89 | 4.54 |
| Micronesia (Federated States of) | 1.26 | 1.69 | 0.90 | 3.98 | 5.13 | 3.02 |
| Monaco | 2.03 | 3.04 | 1.26 | 4.88 | 6.69 | 3.41 |
| Mongolia | 0.89 | 1.25 | 0.58 | 3.03 | 3.71 | 2.43 |
| Montenegro | 1.00 | 1.47 | 0.60 | 3.01 | 3.80 | 2.41 |
| Morocco | 1.68 | 2.20 | 1.19 | 5.08 | 6.83 | 3.76 |
| Mozambique | 0.74 | 1.03 | 0.51 | 2.80 | 3.84 | 1.98 |
| Myanmar | 1.15 | 1.61 | 0.77 | 4.69 | 6.08 | 3.49 |
| Namibia | 0.93 | 1.26 | 0.66 | 3.70 | 4.90 | 2.67 |
| Nauru | 1.23 | 1.64 | 0.88 | 4.74 | 6.46 | 2.95 |
| Nepal | 1.13 | 1.59 | 0.78 | 3.05 | 3.93 | 2.24 |
| Netherlands | 1.85 | 2.67 | 1.18 | 3.49 | 4.87 | 2.56 |
| New Zealand | 0.65 | 0.90 | 0.44 | 2.76 | 3.23 | 2.42 |
| Nicaragua | 1.88 | 2.57 | 1.31 | 3.73 | 4.85 | 2.81 |
| Niger | 0.57 | 0.81 | 0.38 | 2.36 | 3.34 | 1.64 |
| Nigeria | 0.72 | 0.95 | 0.51 | 3.03 | 4.68 | 2.04 |
| Niue | 1.47 | 2.13 | 0.97 | 4.13 | 5.51 | 3.12 |
| North Macedonia | 1.07 | 1.57 | 0.67 | 2.57 | 3.32 | 1.98 |
| Northern Mariana Islands | 1.54 | 2.25 | 0.97 | 3.92 | 5.04 | 3.02 |
| Norway | 1.49 | 2.03 | 1.03 | 3.31 | 4.36 | 2.58 |
| Oman | 1.47 | 2.08 | 1.04 | 3.49 | 4.52 | 2.69 |
| Pakistan | 1.24 | 1.65 | 0.90 | 3.58 | 4.64 | 2.74 |
| Palau | 2.08 | 2.91 | 1.34 | 5.57 | 7.06 | 4.26 |
| Palestine | 1.04 | 1.45 | 0.72 | 2.37 | 3.11 | 1.79 |
| Panama | 2.30 | 3.21 | 1.56 | 4.49 | 6.19 | 3.30 |
| Papua New Guinea | 0.98 | 1.34 | 0.67 | 2.76 | 3.76 | 2.04 |
| Paraguay | 1.85 | 2.53 | 1.30 | 3.39 | 4.63 | 2.45 |
| Peru | 4.23 | 5.59 | 3.04 | 6.08 | 8.41 | 4.37 |
| Philippines | 1.13 | 1.50 | 0.83 | 4.69 | 5.53 | 3.82 |
| Poland | 1.08 | 1.38 | 0.82 | 2.98 | 3.43 | 2.58 |
| Portugal | 1.89 | 2.71 | 1.22 | 3.98 | 5.57 | 2.94 |
| Puerto Rico | 2.22 | 3.05 | 1.52 | 5.42 | 6.92 | 4.14 |
| Qatar | 1.55 | 2.26 | 1.05 | 3.40 | 4.60 | 2.46 |
| Republic of Korea | 1.34 | 1.95 | 0.82 | 3.88 | 4.86 | 3.18 |
| Republic of Moldova | 1.30 | 1.88 | 0.82 | 2.72 | 3.65 | 2.02 |
| Romania | 1.15 | 1.72 | 0.70 | 4.02 | 4.77 | 3.40 |
| Russian Federation | 1.42 | 1.95 | 0.95 | 3.45 | 4.26 | 2.85 |
| Rwanda | 0.80 | 1.11 | 0.55 | 2.56 | 3.51 | 1.79 |
| Saint Kitts and Nevis | 2.22 | 2.95 | 1.56 | 6.02 | 7.78 | 4.78 |
| Saint Lucia | 2.20 | 3.06 | 1.57 | 6.08 | 7.71 | 4.78 |
| Saint Vincent and the Grenadines | 1.91 | 2.59 | 1.34 | 5.74 | 7.13 | 4.64 |
| Samoa | 1.07 | 1.51 | 0.71 | 2.92 | 3.96 | 2.20 |
| San Marino | 1.83 | 2.67 | 1.09 | 2.89 | 4.29 | 1.77 |
| Sao Tome and Principe | 0.97 | 1.33 | 0.66 | 3.79 | 5.35 | 2.73 |
| Saudi Arabia | 1.85 | 2.54 | 1.29 | 8.97 | 11.58 | 6.83 |
| Senegal | 0.71 | 0.97 | 0.47 | 3.36 | 5.02 | 2.32 |
| Serbia | 0.93 | 1.37 | 0.57 | 2.52 | 3.20 | 2.03 |
| Seychelles | 1.68 | 2.39 | 1.11 | 8.08 | 9.74 | 6.80 |
| Sierra Leone | 0.65 | 0.91 | 0.44 | 2.90 | 4.32 | 1.96 |
| Singapore | 1.34 | 1.92 | 0.86 | 2.90 | 3.86 | 2.16 |
| Slovakia | 1.04 | 1.52 | 0.62 | 2.81 | 3.56 | 2.27 |
| Slovenia | 1.20 | 1.85 | 0.68 | 2.50 | 3.37 | 1.88 |
| Solomon Islands | 1.29 | 1.73 | 0.92 | 4.00 | 5.29 | 3.02 |
| Somalia | 0.77 | 1.06 | 0.53 | 2.47 | 3.48 | 1.70 |
| South Africa | 1.05 | 1.39 | 0.77 | 3.99 | 4.82 | 3.28 |
| South Sudan | 0.75 | 1.04 | 0.50 | 2.77 | 3.67 | 2.05 |
| Spain | 2.23 | 3.37 | 1.32 | 4.34 | 6.13 | 3.16 |
| Sri Lanka | 1.31 | 1.92 | 0.86 | 3.90 | 5.21 | 2.80 |
| Sudan | 1.11 | 1.47 | 0.79 | 3.11 | 4.20 | 2.24 |
| Suriname | 1.72 | 2.36 | 1.18 | 4.65 | 6.04 | 3.52 |
| Sweden | 1.61 | 2.19 | 1.10 | 3.04 | 4.13 | 2.25 |
| Switzerland | 1.82 | 2.60 | 1.14 | 3.17 | 4.57 | 2.19 |
| Syrian Arab Republic | 1.35 | 1.95 | 0.89 | 4.52 | 6.04 | 3.39 |
| Taiwan (Province of China) | 1.97 | 2.58 | 1.47 | 6.73 | 7.81 | 5.77 |
| Tajikistan | 0.75 | 1.06 | 0.50 | 2.83 | 3.51 | 2.21 |
| Thailand | 1.56 | 2.22 | 1.03 | 7.50 | 9.57 | 6.01 |
| Timor-Leste | 0.82 | 1.14 | 0.54 | 2.72 | 3.49 | 2.05 |
| Togo | 0.77 | 1.04 | 0.52 | 3.81 | 5.52 | 2.56 |
| Tokelau | 1.23 | 1.72 | 0.80 | 3.43 | 4.59 | 2.58 |
| Tonga | 1.03 | 1.44 | 0.66 | 2.68 | 3.70 | 1.98 |
| Trinidad and Tobago | 2.04 | 2.72 | 1.44 | 5.30 | 6.92 | 4.05 |
| Tunisia | 1.54 | 2.14 | 1.06 | 4.01 | 5.43 | 3.02 |
| Turkey | 1.50 | 2.08 | 1.00 | 4.11 | 5.22 | 3.22 |
| Turkmenistan | 0.87 | 1.27 | 0.56 | 3.31 | 4.19 | 2.50 |
| Tuvalu | 1.18 | 1.64 | 0.81 | 3.81 | 4.93 | 2.85 |
| Uganda | 0.74 | 1.01 | 0.50 | 2.20 | 3.00 | 1.51 |
| Ukraine | 1.72 | 2.41 | 1.17 | 4.43 | 5.70 | 3.32 |
| United Arab Emirates | 3.30 | 4.56 | 2.38 | 4.82 | 6.70 | 3.33 |
| United Kingdom | 1.95 | 2.64 | 1.36 | 4.09 | 5.23 | 3.22 |
| United Republic of Tanzania | 0.81 | 1.08 | 0.57 | 2.40 | 3.26 | 1.75 |
| United States of America | 1.00 | 1.23 | 0.81 | 3.56 | 3.94 | 3.23 |
| United States Virgin Islands | 3.04 | 4.09 | 2.25 | 7.59 | 10.01 | 5.74 |
| Uruguay | 1.08 | 1.60 | 0.66 | 3.18 | 3.94 | 2.65 |
| Uzbekistan | 0.87 | 1.22 | 0.56 | 2.22 | 2.86 | 1.73 |
| Vanuatu | 1.19 | 1.60 | 0.82 | 3.94 | 5.10 | 2.97 |
| Venezuela (Bolivarian Republic of) | 2.37 | 3.30 | 1.62 | 5.61 | 7.51 | 4.22 |
| Viet Nam | 1.61 | 2.26 | 1.14 | 5.37 | 7.14 | 4.08 |
| Yemen | 1.02 | 1.36 | 0.76 | 3.00 | 4.09 | 2.12 |
| Zambia | 0.84 | 1.16 | 0.58 | 2.60 | 3.40 | 1.96 |
| Zimbabwe | 0.86 | 1.16 | 0.60 | 4.38 | 5.99 | 3.15 |
