# Supplementary material for: Temporal and geographical dynamics of early-onset Parkinson’s disease burden: insights from the Global Burden of Disease Study 2021
Source: Front Neurol. 2025 Jan 30;16:1473548. doi: 10.3389/fneur.2025.1473548 (PMC11821659; doi:10.3389/fneur.2025.1473548)
Supplement: Supplementary file 2 [file Table_2.DOCX]

Supplementary Table 2 Alterations in Incidences number based on the population-level determinants and causes between 1990 and 2021

|  |  | change due to population-level determinants | | | % contribute to the total changes | | |
| --- | --- | --- | --- | --- | --- | --- | --- |
| Location | Overall difference | Aging | Population | Epidemiological change | Aging | Population | Epidemiological change |
| Central Sub-Saharan Africa | 393.67 | 16.606 | 337.096 | 39.969 | 4.22 | 85.63 | 10.15 |
| Eastern Sub-Saharan Africa | 1125.87 | 45.523 | 1034.453 | 45.895 | 4.04 | 91.88 | 4.08 |
| Western Sub-Saharan Africa | 1147.26 | 8.247 | 1017.662 | 121.348 | 0.72 | 88.7 | 10.58 |
| Southern Sub-Saharan Africa | 231.59 | 45.082 | 164.937 | 21.57 | 19.47 | 71.22 | 9.31 |
| North Africa and Middle East | 7632.11 | 450.211 | 5027.807 | 2154.096 | 5.9 | 65.88 | 28.22 |
| High-income Asia Pacific | 40.78 | 77.328 | -120.307 | 83.76 | 189.62 | -295.01 | 205.39 |
| Central Asia | 186.85 | 57.014 | 139.257 | -9.417 | 30.51 | 74.53 | -5.04 |
| East Asia | 25782.01 | 4312.309 | 1938.167 | 19531.537 | 16.73 | 7.52 | 75.76 |
| Southeast Asia | 2421.53 | 543.004 | 1487.886 | 390.636 | 22.42 | 61.44 | 16.13 |
| South Asia | 20466.91 | 294.881 | 14176.482 | 5995.545 | 1.44 | 69.27 | 29.29 |
| Central Europe | -60.32 | 61.184 | -81.794 | -39.714 | -101.43 | 135.6 | 65.84 |
| Eastern Europe | -19.19 | 147.788 | -164.187 | -2.787 | -770.13 | 855.59 | 14.52 |
| Western Europe | 531.34 | 152.995 | -28.454 | 406.797 | 28.79 | -5.36 | 76.56 |
| Tropical Latin America | 1567.54 | 285.265 | 778.706 | 503.566 | 18.2 | 49.68 | 32.12 |
| Southern Latin America | 155.66 | 16.524 | 82.231 | 56.9 | 10.62 | 52.83 | 36.55 |
| Central Latin America | 1698.52 | 283.737 | 934.428 | 480.359 | 16.7 | 55.01 | 28.28 |
| Andean Latin America | 988.78 | 132.39 | 509.483 | 346.91 | 13.39 | 51.53 | 35.08 |
| Caribbean | 185.26 | 33.525 | 94.409 | 57.327 | 18.1 | 50.96 | 30.94 |
| High-income North America | -92.77 | -84.78 | 224.255 | -232.246 | 91.39 | -241.73 | 250.35 |
| Oceania | 47.79 | 4.464 | 38.196 | 5.135 | 9.34 | 79.92 | 10.74 |
| Australasia | 38.94 | -0.159 | 17.442 | 21.653 | -0.41 | 44.79 | 55.61 |
| Global | 51197.2 | 5108.924 | 20330.168 | 25758.104 | 9.98 | 39.71 | 50.31 |
