# Supplementary material for: Temporal and geographical dynamics of early-onset Parkinson’s disease burden: insights from the Global Burden of Disease Study 2021
Source: Front Neurol. 2025 Jan 30;16:1473548. doi: 10.3389/fneur.2025.1473548 (PMC11821659; doi:10.3389/fneur.2025.1473548)
Supplement: Supplementary file 3 [file Table_3.DOCX]

Supplementary Table 3 Alterations in DALYs number based on the population-level determinants and causes between 1990 and 2021

|  |  | change due to population-level determinants | | | % contribute to the total changes | | |
| --- | --- | --- | --- | --- | --- | --- | --- |
| Location | Overall difference | Aging | Population | Epidemiological change | Aging | Population | Epidemiological change |
| Central Sub-Saharan Africa | 1161.85 | 90.448 | 1092.944 | -21.545 | 7.78 | 94.07 | -1.85 |
| Eastern Sub-Saharan Africa | 3120.58 | 235.481 | 3247.196 | -362.092 | 7.55 | 104.06 | -11.6 |
| Western Sub-Saharan Africa | 5017.35 | 79.26 | 4702.39 | 235.7 | 1.58 | 93.72 | 4.7 |
| Southern Sub-Saharan Africa | 1027.69 | 215.424 | 630.898 | 181.364 | 20.96 | 61.39 | 17.65 |
| North Africa and Middle East | 17880.42 | 1982.035 | 17298.762 | -1400.38 | 11.08 | 96.75 | -7.83 |
| High-income Asia Pacific | -153.13 | 316.972 | -366.919 | -103.183 | -207 | 239.61 | 67.38 |
| Central Asia | 499.72 | 206.51 | 424.653 | -131.439 | 41.33 | 84.98 | -26.3 |
| East Asia | 13581.65 | 9618.261 | 4312.192 | -348.802 | 70.82 | 31.75 | -2.57 |
| Southeast Asia | 9405.01 | 3183.514 | 5896.079 | 325.417 | 33.85 | 62.69 | 3.46 |
| South Asia | 47363.67 | 1284.872 | 39086.008 | 6992.785 | 2.71 | 82.52 | 14.76 |
| Central Europe | -441.43 | 227.727 | -255.304 | -413.851 | -51.59 | 57.84 | 93.75 |
| Eastern Europe | 187.54 | 445.569 | -394.245 | 136.213 | 237.59 | -210.22 | 72.63 |
| Western Europe | 210.47 | 475.173 | -61.325 | -203.374 | 225.77 | -29.14 | -96.63 |
| Tropical Latin America | 3039.17 | 831.035 | 1706.228 | 501.911 | 27.34 | 56.14 | 16.51 |
| Southern Latin America | 271.97 | 57.255 | 250.663 | -35.946 | 21.05 | 92.17 | -13.22 |
| Central Latin America | 3571.04 | 906.849 | 2383.193 | 280.993 | 25.39 | 66.74 | 7.87 |
| Andean Latin America | 1170.41 | 243.812 | 838.555 | 88.042 | 20.83 | 71.65 | 7.52 |
| Caribbean | 475.01 | 110.044 | 249.664 | 115.304 | 23.17 | 52.56 | 24.27 |
| High-income North America | 368.21 | -204.861 | 682.452 | -109.383 | -55.64 | 185.34 | -29.71 |
| Oceania | 120.92 | 15.378 | 116.654 | -11.114 | 12.72 | 96.47 | -9.19 |
| Australasia | 114.34 | 0.073 | 63.989 | 50.275 | 0.06 | 55.96 | 43.97 |
| Global | 76427.6 | 15804.116 | 53782.734 | 6840.748 | 20.68 | 70.37 | 8.95 |
